# Unveiling the Role of Theory of Mind: Neural Response to Emotional Stimuli in Context

**DOI:** 10.1007/s42761-025-00293-1

**Published:** 2025-02-26

**Authors:** Brigitte Biró, Renáta Cserjési, Natália Kocsel, Attila Galambos, Kinga Gecse, Lilla Nóra Kovács, Dániel Baksa, Dóra Dobos, Gabriella Juhász, Gyöngyi Kökönyei

**Affiliations:** 1 Doctoral School of Psychology, ELTE Eötvös Loránd University, Budapest, Hungary; 2 NAP3.0-SE Neuropsychopharmacology Research Group, Hungarian Brain Research Program, Semmelweis University, Budapest, Hungary; 3 Institute of Psychology, ELTE Eötvös Loránd University, Budapest, Hungary; 4 Department of Clinical Psychology, Semmelweis University, Budapest, Hungary; 5 Department of Pharmacodynamics, Faculty of Pharmaceutical Sciences, Semmelweis University, Budapest, Hungary; 6 Doctoral School of Mental Health Sciences, Semmelweis University, Budapest, Hungary; 7 Department of Personality and Clinical Psychology, Institute of Psychology, Faculty of Humanities and Social Sciences, Pazmany Peter Catholic University, Budapest, Hungary

**Keywords:** Theory of mind, Context, Emotional processing, fMRI, Self-to-other model

## Abstract

The recent literature on theory of mind (ToM) aims to identify situations that necessitate a greater reliance on ToM. The self-to-other model of empathy proposes that when there is a discrepancy between affective and contextual information, the ToM system is activated more strongly to assist in the classification of the emotional state. In order to test this aspect of the theory, the study aimed to examine the neural response to emotional stimuli in context. Non-congruent images depict natural scenarios where there is a mismatch between the valence of facial expressions of one of the protagonists and the overall context, when considered in isolation. In contrast, congruent images are those in which there is no such discrepancy. The fMRI data of 53 healthy participants (31 females; mean age, 25.23 ± 5.09) were analysed using a pre-defined ToM mask. The results align with the prediction that non-congruent images will recruit the ToM system to a greater degree. Additionally, we found activations in areas implicated in emotion processing and control functions in response to non-congruent images relative to congruent ones. The results indicate that the emotional processing of complex social situations is supported by multiple processes. Consequently, identifying the sources of alterations in emotion processing and social cognition is crucial for understanding individual differences in neurotypical and clinical populations.

**Supplementary Information:**

The online version contains supplementary material available at 10.1007/s42761-025-00293-1.

Empathy and theory of mind (ToM) are socio-cognitive skills crucial for social and emotional functioning (Goldstein & Winner, 2012; Kanske, 2018). Empathy involves vicariously experiencing others’ emotional states (Davis, 1983), while ToM (or mentalizing) encompasses inferring mental states such as beliefs, desires, intentions, and emotions in others (Frith & Frith, 2006; Premack & Woodruff, 1978). To comprehend or experience others’ feelings, we integrate various informational cues, such as facial expressions, postural cues, semantic information, and situational cues, along with emotional and social knowledge about the context (Wieser & Brosch, 2012). Thus, in social situations, we not only decode affective cues, but also utilize situational cues and emotional/social knowledge to grasp the overall context (Sabbagh, 2004). These two types of cues are processed by distinct neural circuits (Bird & Viding, 2014) and interact with each other, moderating their effects. For instance, our understanding of a situation, such as a wedding, shapes how we interpret person-level information, such as observing crying parents. Conversely, person-level cues, like seeing a crying person, can affect understanding of the situation—such as when the person is reading a letter. Therefore, understanding another person’s mental state is a complex process that may benefit from a more granular examination of its constituent components. It has been argued that at least three processes, which are mainly parallel and interdependent (Spunt & Adolphs, 2019), contribute to the understanding of others’ emotions. These processes are as follows: (1) detecting and attending to relevant emotional cues; (2) inferring the emotional state being experienced by the target; and (3) identifying the causes of these feelings (i.e., the reason behind the emotion being felt).

To further specify the complex phenomenon of emotion understanding, Houlihan et al. (2023) present a comparative analysis of two models for understanding emotions. According to the emotion recognition model, it would be sufficient for the observer to rely on the emotional expressions of others to make accurate inferences about their emotions or intentions. In contrast, the reasoning model posits that emotions are not directly discernible; rather, they are inferred based on the identification of the causal explanation that best accounts for the co-occurrence of events, emotions, and expressions. In this model, it is possible that the emotions predicted based on a given event/situation may reflect disparate information than those attributed based on the expressions of other individuals being in that situation. To illustrate, crying is an emotional expressive behavior commonly associated with sadness (emotion). However, when the same expression is observed in the context of a wedding (situation), it is more likely to be interpreted as a sign of joy (see Fig. 1). Thus, the perception of an emotional expression is context-dependent (Fernández-Dols & Carroll, 1997). Furthermore, context can lead to a categorical shift in emotion perception (e.g., from sadness to joy, as illustrated in the previous example), which is supported by empirical evidence (Aviezer et al., 2012). A recent meta-analysis (Durán & Fernández-Dols, 2021) also indicated that the identification of basic emotions is imperfect if the observer relies solely on the facial signals. The mean co-occurrence of full expressions in facial signals and basic emotion categories (happiness, sadness, anger, disgust, fear, and surprise) across empirical studies that used the Facial Actions Coding systems was only .13. This increased to .23 when studies that used alternative coding systems or partial facial expressions were included in the meta-analysis. Consequently, these meta-analytic results suggest that the prevailing notion—namely, that an individual’s emotional state can be readily discerned from their facial expressions—is not well supported by empirical data, and thus, the emotion recognition model lacks strong empirical backing (for a comprehensive review, see Barrett et al., 2019).

**Fig. 1:**
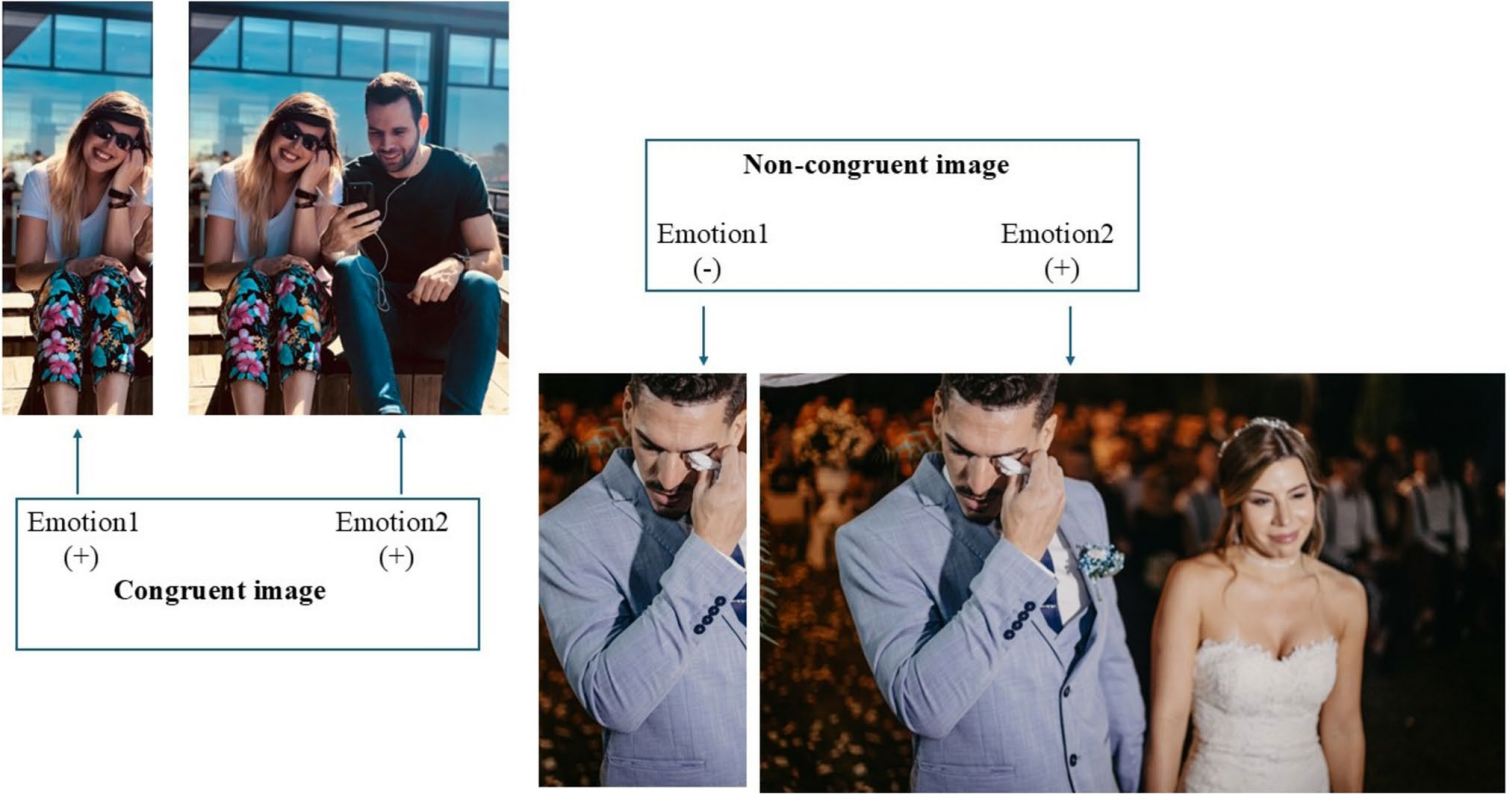
Examples for congruent and non-congruent images. Both images are free to use (pexels.com, photographers: Dziubi Steenbergen and Jonathan Nenemann). Note that Emotional Shifting Task does not contain these two images (Biro et al., 2021)

Affective cues and situation cues provide information to the ToM system, aiding in the representation of others’ mental states. All the three types of information contribute to the classification of the social target’s emotional state (Bird & Viding, 2014). Consequently, in this approach—similar to other influential theories (e.g., Frith & Frith, 2006; for a review, see Mitchell & Phillips, 2015)—the perception of emotion serves as a precursor to ToM. The perception of emotion heavily relies on the context itself, as we naturally infer that the context or event influences the target’s emotional state, as reflected in their emotional expressions (Saxe & Houlihan, 2017). However, it remains uncertain whether processing certain emotional stimuli within a given context necessitates a greater reliance on ToM than processing others.

A key focus in recent ToM literature is understanding the factors that influence the extent to which an individual represents the mental state of others in a given situation (Conway & Bird, 2018). In other words, are there specific situations that demand a stronger reliance on ToM than others (Bio et al., 2018; Conway & Bird, 2018)? For example, decoding self-conscious emotions, such as pride and embarrassment, as opposed to basic emotions like anger and surprise, and perceiving the overall social situation in which protagonists are involved, resulted in greater activations of certain regions (e.g., the right temporoparietal junction) of the neural ToM system (Caillaud et al., 2020).

We hypothesize that emotional stimuli within a given context will evoke greater activation of the ToM system when the emotions predicted based on the event or situation reflect different information than those attributed based on the expressions of individuals involved in that situation. For example, witnessing a person crying often leads to the assumption that they are sad. However, observing a crying person in a specific context may yield different emotion attributions. For instance, witnessing someone crying at a wedding might lead us to infer that they are experiencing joy. We propose that this scenario engages the ToM system more strongly than observing a person crying at a funeral, where our emotion attribution aligns with the conventional understanding of sadness. This hypothesis is grounded in the self-to-other model of empathy (Bird & Viding, 2014), in which the ToM system is proposed to be more strongly activated when there is a discrepancy between the output of the Affective Cue Classification System, representing affective cues, and the Situation Understanding System, representing situational cues.

To test this aspect of the theory, the present study utilized the Emotional Shifting Task (Biro et al., 2022; Lacroix et al., 2022) in the MR scanner. This task includes different sets of socially relevant visual stimuli depicting everyday life situations (see Fig. 1). In some images, there is congruence between affective and situational cues, indicating alignment between the outputs of the Affective Cue Classification System and the Situational Cue Classification System. For example, a smiling girl (Emotion1 image, positive valence) enjoying music with someone else (Emotion2 image, positive context) allows for the straightforward conclusion that the girl is experiencing positive affect. In contrast, other images show a discrepancy between the facial expression of (at least) one of the protagonists and the overall context. For instance, there is a discrepancy between the affective cue provided by a crying man (Emotion1 image, negative valence) and the situational cue indicating that it is his wedding (Emotion2 image, positive valence). However, crying on one’s wedding day is socially acceptable and the observer can easily infer that the man is emotionally moved and happy. In contrast, merely observing the face of the crying man would lead to a vastly different emotional attribution, supporting the reasoning model. Thus, within the framework of the reasoning model (see above), the non-congruent images in our task can be characterized by the presence of conflicting perceptual and conceptual information, whereas congruent images are characterized by the absence of such conflicting information (see Fig. 1). Accordingly, an Emotion2 image was considered congruent if the overall valence of Emotion1 and Emotion2 was identical (e.g., both positive or both negative). Images were classified as non-congruent if the valence of Emotion1 and Emotion2 differed (e.g., if Emotion1 was positive and Emotion2 negative, or vice versa). Thus, in line with the self-to-other model, we propose that these non-congruent images would necessitate greater involvement of the ToM system compared to congruent ones, in order to comprehend the overall situation or become emotionally involved. The aforementioned images are labelled as non-congruent, illustrating that, in the absence of contextual information, affective cues would lead to different emotional attributions.

Meta-analyses (Molenberghs et al., 2016; Schurz et al., 2014) indicate that core regions associated with ToM include the anterior and dorsal part of the medial prefrontal cortex (mPFC) and bilateral temporoparietal junction (TPJ), as well as an extended network encompassing the temporal poles, superior temporal gyrus/superior temporal sulcus (STG/STS), and precuneus/posterior cingulate cortex. Findings from a task (Kanske et al., 2015), designed to disentangle empathy and ToM, also support the involvement of ventral TPJ, STG, temporal poles, and anterior and posterior midline regions in mentalizing (Hildebrandt et al., 2021; Tholen et al., 2020).

To test our hypothesis that non-congruent images, compared to congruent images, would require greater involvement of the ToM system, we applied a specific mask of the ToM brain regions (Dufour et al., 2013). Given that other networks and brain areas contribute to the understanding of others’ mental states (da Costa et al., 2022), we also conducted a whole-brain analysis to identify regions outside of the ToM mask. Additionally, we explored whether there are any sex differences in activations to non-congruent vs. congruent images, as previous studies have shown some differences in empathy and ToM (see Christov-Moore et al., 2014; Rochat, 2023). However, findings are mixed and gender differences among adults might be more relevant for self-report data (Baez et al., 2017).

It is important to note that our previous publication (Biro et al., 2022) presented findings based on the Emotional Shifting Task, which addressed a different research question. The primary objective was to examine how context influenced alterations in emotional response. To this end, we conducted a comparative analysis of the BOLD response to images with context (Emotion2) vs. without context (Emotion1). The results demonstrated that images with context engaged a number of brain regions implicated in the perception, attention to, and processing of emotional stimuli. Furthermore, regions such as the temporoparietal junction (TPJ) and the precuneus, which are involved in ToM, were also engaged (Biro et al., 2022). In the present study, we conducted a more detailed analysis of the responses to Emotion2 images, which were categorized as either congruent or non-congruent. This allowed us to investigate whether specific attributes of images with context differently recruit the ToM network. It is also important to note that the current study includes data from 53 participants, 31 of whom overlap with the data presented in the previous study.

## Method

### Participants

Fifty-nine healthy volunteers participated in our study. Six participants were excluded from the analysis due to excessive motion in the scanner or incomplete data; thus, data from 53 participants were used in the analyses (31 females and 22 males [mean age, 25.23; *SD*, 5.09; age range, 20–49 years]). All participants were Hungarian (and Caucasian), 28 participants had a high school degree, 24 reported having a graduate degree, and 1 had a professional qualification. The subjects were right-handed, assessed by a standardized handedness questionnaire (Oldfield, 1971), and had normal or corrected-to-normal vision. Recruitment occurred through a social media site (Facebook) and journal advertisements. Participants were examined by a senior psychiatrist and a neurologist and were excluded from the research with any history of medical, psychiatric, and neurological disorder diagnosis. Patients with headaches (migraine, tension-type, and medication overuse) were also included in this study. However, for the purposes of this study, only the results pertaining to the healthy control subgroup are presented and discussed.

#### Ethics Approval Statement

The study was approved by the Scientific and Research Ethics Committee of the Medical Research Council (Hungary). Each participant gave a written informed consent before entering the study in accordance with the Declaration of Helsinki.

### Psychological Task: The Emotional Shifting Task (EST)

**Fig. 2:**
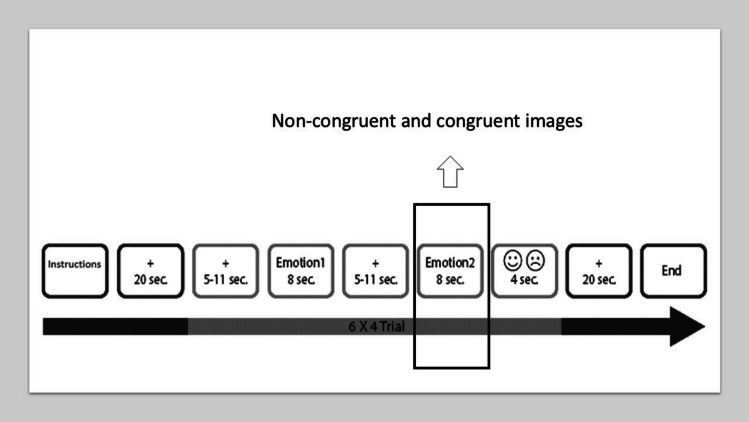
The design of Emotional Shifting Task

The EST (Biro et al., 2021, 2022) was originally designed to measure the ability of emotional flexibility, by using visual stimuli and picturing social, everyday life situations. The task consists of trials of image pairs. In most cases, the cropped image (Emotion1) expressed emotion primarily through facial expression and/or posture. It is then followed by the second image of the pair, which presents the whole image. The additional context can change the entire interpretation of the image by changing its valence and/or arousal. After each pair, a happy and a sad smiley was shown on the response screen (design and stimuli example in Fig. 2). Participants were asked to decide which of the two smileys describes better their emotions after observing the set of stimuli. We opted to utilize emojis within the scanner, thereby mimicking the two endpoints of valence ratings as observed in the Self-Assessment Manikin (Lang et al., 1997).

The first image was either a negative or a positive, whereas the second, whole image added the background context that changed the valence of the first image, either to a positive or to a negative (P– > N or N– > P) or did not change the valence (P– > P or N– > N). In this study, the former images were classified as non-congruent, whereas the latter were classified as congruent (see Fig. 1). Each condition consisted of six pairs of images, shown in a pseudo-random order. Image pairs where the valence has not changed are from the International Affective Picture System (Lang et al., 1997). Their identification numbers were 1340, 2091, 2141, 2205, 2216, 2340, 2530, 2700, 6242, 6838, 8497, and 9050. The other images were chosen from the internet, were free to use, and were piloted for valence, arousal, and dominance (Biro et al., 2021). In this analysis, we focused on only the whole images with background: congruent images were the IAPS pictures, while the non-congruent ones were chosen from the internet (for more details about the images, see Biro et al., 2022).

In the scanner, participants were asked to view the images and emotionally engage with the depicted situation (they had previously completed a brief practice task). To measure baseline brain activity, a white fixation cross was presented on a black screen for 20 s at the beginning of the session. This was repeated at the end of the task, as well. Each emotional stimuli were presented for 8 s. This setup was based on laboratory pilot studies and previous research in which labels were used to influence emotion processing and evaluation (Deak et al., 2017). Our approach was also informed by studies on cognitive reappraisal, such as those by Ochsner et al. (2002, 2004; Denny et al., 2015), who typically presented images for reappraisal for 8 s. The conscious reappraisal process is considered effortful (Braunstein et al., 2017). Therefore, we anticipated that processing and evaluating images depicting naturalistic emotional scenes (e.g., a couple cycling together or a girl being laughed at by others) as either negative or positive would be a less effortful process (compared to conscious reappraisal). This is because contextual information is integrated with facial and postural cues during the early phase of emotion perception (Hietanen & Astikainen, 2013). To minimize artifacts caused by expectations, fixational crosses were presented with varying durations (ranging from 5 to 11 s, with a mean of 8 s) before each emotional stimulus. At the end of each trial, a response screen was presented for 4 s. For stimulus presentations, E-Prime 2.0 Software was used (Psychology Software Tools, Inc., Pittsburgh, PA, USA).

Following the scanning session, participants rated the valence and arousal of all images on a 7-point Likert scale, with 1 representing “very unpleasant” (for valence) or “calm” (for arousal) and 7 representing “very pleasant” (for valence) or “excited” (for arousal). With regard to the number of protagonists depicted in the images, four of the non-congruent images feature two protagonists, while five of the congruent images also show two protagonists. The remaining images in both categories show a greater number of protagonists, with three or more.

### FMRI Acquisition

Functional MRI data acquisition was registered on a SIEMENS MAGNETOM Prisma syngo MR D13D 20 channels headcoil scanner. A series of high-resolution anatomical images were obtained during the first functional imaging session using a T1-weighted 3D TFE sequence with 1 × 1 × 1 mm resolution for the structural data. A BOLD-sensitive T2*-weighted echo-planar imaging sequence was used (TR = 2220 ms, TE = 30 ms, FOV: 222) with 3 mm × 3 mm in-plane resolution and contiguous 3-mm slices providing whole-brain coverage. There were 409 volumes during the task.

### fMRI Data Analyses

#### Preprocessing

For analysing the imaging data, Statistical Parametrical Mapping (SPM12) analysis software package (Wellcome Department of Imaging Neuroscience, Institute of Neurology, London, UK; https://www.fil.ion.ucl.ac.uk/spm/software/spm12/) in Matlab 2016b (Math Works, Natick, MA, USA) was used. The steps of the processing analyses on the functional images were the following: realignment, co-registration to the structural image, segmentation, normalization in Montreal Neurological Institute (MNI) space, and spatial smoothing with an 8-mm full-width half-maximum Gaussian kernel. Lastly, to control for the quality of images, we inspected them visually to exclude poor-quality images.

#### First-Level Model

In the first-level analyses, we used a general linear model (GLM), where blood oxygenation level-dependent (BOLD) hemodynamic responses were modelled. In the event-related single subject analysis fixation screens, Emotion1 images (multiple regressors were used: positive-congruent, positive-incongruent, negative congruent, and negative incongruent details), Emotion2 images (multiple regressors were used: positive-congruent, positive-incongruent, negative congruent, and negative incongruent), and the response screen (happy and sad emojis) were modelled as separate regressors of interest. All the regressors in the GLM were convolved with a hemodynamic response function, the canonical (double gamma) response function. High-pass temporal filtering with a cut-off of 128 s was included in the model in order to eliminate all the effects of low-frequency physiological noise; furthermore, serial correlations in data series were assessed using an autoregressive AR (1) model. The Artifact Detection Tools (ART www.nitrc.org/projects/artifact_detect/) and six motion parameters were used as regressors of no interest in the fMRI model to identify motion outliers (threshold of global signal > 3 SD and motion > 1 mm).

To investigate the recruitment of the ToM system when incongruence occurs between informational cues (e.g., facial expressions) and contextual information (Bird & Viding, 2014), we employed a contrast that included only the second set of images, i.e., the whole images (i.e., non-congruent ones minus congruent ones) (Emotion2, see Fig. 2).

Note that in our previous publication (Biro et al., 2022), we analysed a different contrast. The main question was how context drove changes in the emotional response; therefore, we used an image with context vs. without context contrast. This was done upon completion of our previous project with a sample size of *N* = 31. The current study presents data from 53 participants, of which 31 overlap with the data presented in the previous study.

#### Second-Level Analyses

To investigate the group-level effects, the individual contrasts of non-congruent images vs. congruent images were entered into a second-level one-sample *t*-test in SPM. The resulting *t*-statistic image was corrected for multiple comparisons using voxel-level family-wise error (FWE) correction at a threshold of *p* < .05, with a minimum cluster size of *k* voxels, where the *k*-extent was determined based on the voxel-level threshold to control for false positives.

We applied a specific mask of the theory of mind-brain regions (Dufour et al., 2013); the thresholded maps were retrieved from https://saxelab.mit.edu/use-our-theory-mind-group-maps. The mask included seven regions: the dorsomedial (DMPFC), ventromedial (VMPFC) and middle medial prefrontal cortex (MMPFC), the left and right temporoparietal junction (LTPJ, RTPJ), the precuneus, and the right superior temporal sulcus (rSTS).

 To anatomically identify the activated clusters, automated anatomical labelling atlas (aal) was used (Rolls et al., 2020; Tzourio-Mazoyer et al., 2002), and to visualize statistical maps, the MNI 152 template brain provided in MRIcroGL was used (http://www.mccauslandcenter.sc.edu/mricrogl/) (Rorden & Brett, 2000). In addition, we also explored whether there are any sex differences in activations to non-congruent vs. congruent images using an independent sample *t*-test.

Main fMRI contrast maps are fully available at https://osf.io/zqwub/.

### Statistical Analysis of Self-Report, Reaction Times, and Post-Scan Data

The analysis of demographic and behavioral data was conducted using SPSS version 29.0 (IBM SPSS, IBM Corp, Armonk, NY, USA). Paired *t*-tests and a 2 × 2 repeated measures of ANOVA were performed on the reaction times collected during the fMRI scan. The same methodology was employed for the analysis of ratings of valence and arousal.

### Low-Level Visual Complexity

In order to eliminate the possibility that the neural response to the two types of images is contingent on their visual complexity, three indices of computational visual complexity were calculated (Madan et al., 2018). The term “edge density” refers to the ratio of pixels representing edges to the total number of pixels in the image. Essentially, it quantifies the extent to which boundaries are present within the image, which can be an indicator of image structure and detail. The measure of feature congestion quantifies the degree of visual clutter in an image, considering multiple factors such as color variation, luminance contrast, and orientation. It reflects how densely features (e.g., edges, textures) are packed in the image. Subband entropy is the organization of the image by measuring the level of unpredictability or randomness within spatial repetitions of hue, luminance, and size (spatial frequency), using Shannon’s entropy. A higher entropy value suggests a more disordered or complex distribution of these features across the image. For the calculations, a Python script was used with the OpenCV (version 4.10.0) and skimage (version .24.0) libraries. The cv2.Canny function was employed to calculate edge density, the cv2.Sobel function was used to assess feature congestion based on gradient magnitude, and the pywt.dwt2 function with Discrete Wavelet Transform (DWT) was applied to compute subband entropy.

## Results

### Low Level of Visual Complexity

The results of the independent *t*-tests indicated that there were no statistically significant differences in visual complexity between the two types of images (see Table 1).

**Table 1:** Mean (SD) values for the computational measure indices for non-congruent and congruent images

Measures	Image category		*t*	*p*
	Non-congruent (*N* = 12)	Congruent (*N* = 12)		
Computational visual complexity				
Edge density	.08 (.03)	.11 (.05)	1.934	.066
Feature congestion	85.56 (18.57)	104.38 (37.26)	1.565	.132
Subband entropy	4.91 (0.82)	5.14 (0.01)	0.604	.552

### Behavioral Data During EST in the Scanner

During the EST, “accuracy” of the answers of the participants (whether they picked the expected smiley/emoji shown on the screen) and reaction times were collected. The accuracy rate was 94.1% (range, 62.5–100.0%).

A 2 (congruent vs. non-congruent) × 2 (positive vs. negative) repeated measures analysis of variance (ANOVA) was used to test whether there is a difference in the behavioral responses (reaction time) to the emojis. Results show that both factors are associated with the reaction times. Specifically, participants exhibited a longer reaction times (*F*(1, 52) = 7.560, *p* = .008) to non-congruent trials (*M* = 1000.78, *SD* = 298.31) compared to congruent ones (*M* = 934.84, *SD* = 276.77) and to negative (*M* = 1035.46, *SD* = 312.24) compared to positive images (*M* = 901.99, *SD* = 269.76) (*F*(1, 52) = 22.218, *p* < .001). Nevertheless, no significant interaction was observed between the two within-subject factors (*F*(1, 52) = 1.599, *p* = .212). However, the post hoc analysis showed that subjects responded to the response screen more slowly in trials involving negative and non-congruent stimuli compared to the other three types of trials (see Table 2), confirming our previous results using a subsample of this study (Biro et al., 2022).

**Table 2:** Arousal and valence ratings of Emotion2 images in the post-scan task and reaction time to response screen in the scanner

	Arousal Mean (*SD*)	Valence Mean (*SD*)	Reaction time Mean (*SD*)
Congruent/positive	4.93 (1.04)	6.11 (0.73)	880.44 (269.08)
Congruent/negative	5.09 (0.93)	2.04 (0.79)	989.24 (328.45)
Non-congruent/positive	5.01 (1.13)	5.78 (0.77)	923.77 (320.21)
Non-congruent/negative	5.05 (1.06)	2.17 (0.69)	1081.31 (332.83)

### Post-Scan Ratings

There was no difference in overall arousal between the congruent and incongruent images (*t*(52) = 0.231, *p* = .819; non-congruent images: *M* = 5.03, *SD* = 1.02; congruent images: *M* = 5.01, *SD* = .79) according to the paired *t*-test results. As both types of images consist of six negative and six positive images, in the subsequent step, a 2 (congruent vs. non-congruent images) × 2 (positive vs. negative images) repeated measures of ANOVA were conducted. The results demonstrated that neither the main effect of congruency (*F*(1,52) = 0.053, *p* = .819) nor the valence category (*F*(1,52) = 0.964, *p* = .331) was significant. Furthermore, the interaction between these within-subject factors was also non-significant (*F*(1, 52) = 0.406, *p* = .527).

With regard to the valence ratings of the images, a paired *t*-test was initially conducted, which indicated no significant difference (*t*(52) = 1.245, *p* = .819; non-congruent images: *M* = 3.98, *SD* = .49); congruent images: *M* = 4.08, *SD* = .53). However, as the image set included both positive and negative images within the congruent and non-congruent categories, a 2 (congruent vs. non-congruent images) × 2 (positive vs. negative images) repeated measures of ANOVA were conducted in the subsequent step. It was expected that the positive/negative image category would have a significant main effect on valence ratings. Indeed, the positive images were rated higher on a 7-point Likert scale in comparison to the negative images (*F*(1, 52) = 996.019, *p* < .001), while the main effect of congruency proved to be non-significant (*F*(1, 52) = 1.434, *p* = .237). A significant interaction was observed between the two factors (*F*(1, 52) = 7.115, *p* = .010). The univariate analysis demonstrated that negative images were rated similarly, irrespective of whether they were congruent or incongruent (*t*(52) = 0.957, *p* = .343). Conversely, congruent positive images were rated slightly more positively than non-congruent positive images (*t*(52) = 3.679, *p* < .001). However, in both cases, the mean rating was approximately 6 (with a maximum of 7), indicating that both ratings were situated at the highly pleasant end of the scale (Table 2).

### Small-Volume Correction Within the ToM Mask

**Fig. 3:**
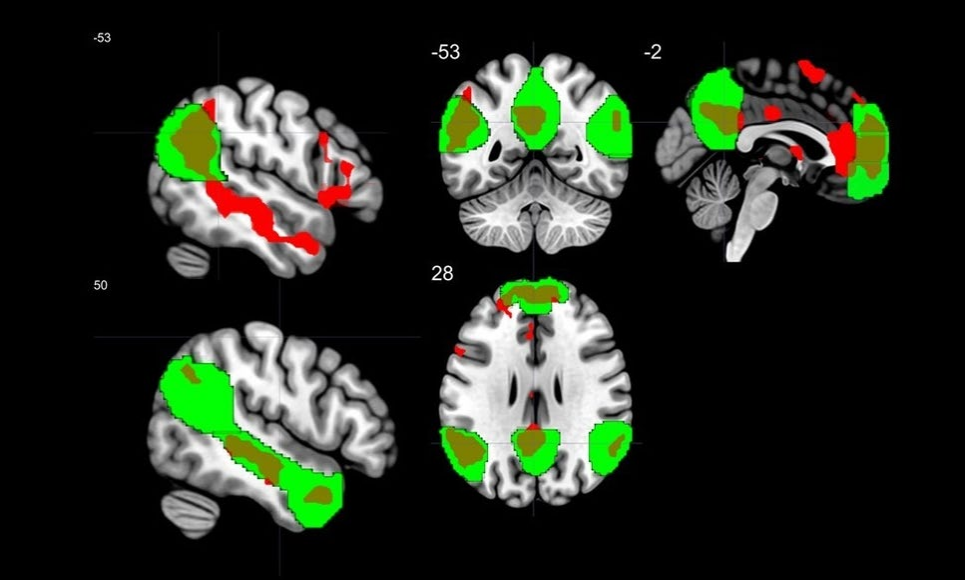
The overlapping areas of the theory of mind (ToM) mask and the whole-brain analysis. Green colored areas are brain regions of the ToM mask (Dufour et al., 2013), whereas red areas are the results of our whole-brain analysis (see Supplementary material, Table S1). The regions that overlap contribute to our main results and are listed in Table 3. ToM mask and statistical map were visualized on the MNI 152 template brain provided in MRIcroGL (http://www.mccauslandcenter.sc.edu/mricrogl/) (Rorden & Brett, 2000)

To test our hypothesis, we implemented a small-volume correction within the ToM mask (Dufour et al., 2013) and found increased activations in all the regions (Table 3 and Fig. 3), namely the dorsomedial (DMPFC), ventromedial (VMPFC) and middle medial prefrontal cortex (MMPFC), the left and right temporoparietal junction (LTPJ, RTPJ), the precuneus, and the right superior temporal sulcus (rSTS) to non-congruent vs. congruent images. The other contrast (congruent vs. non-congruent images) did not produce any significant voxels.

**Table 3:** Activations in theory of mind system to non-congruent vs. congruent images (FWE corrected level of *p* < .05)

Regions		Hemisphere	*T*-value	MNI coordinates		
Regions in AAL	Region in the ToM mask			x	y	z
Medial prefrontal cortex (voxel size: 606)						
Superior frontal gyrus, medial	MMPFC	L	8.121	− 6	59	11
Anterior cingulate cortex, pregenual	MMPFC	L	7.067	− 3	50	8
Anterior cingulate gyrus, pregenual	MMPFC		6.953	0	44	5
Superior frontal gyrus, medial	DMPFC	R	6.663	9	56	26
Middle frontal gyrus	DMPFC	L	6.395	− 24	47	32
Superior frontal gyrus, medial	DMPFC	L	6.305	− 9	56	26
Anterior cingulate cortex pregenual	VMPFC	L	6.256	− 3	38	− 7
Superior frontal gyrus, medial orbital	VMPFC	L	6.102	− 3	53	− 7
Superior frontal gyrus	DMPFC	L	5.804	− 9	53	35
Superior frontal gyrus	DMPFC	L	5.674	− 18	59	23
Left temporoparietal junction (cluster size: 411)						
Angular gyrus	LTPJ	L	7.976	− 45	− 67	44
Angular gyrus	LTPJ	L	7.619	− 51	− 55	32
Inferior parietal gyrus	LTPJ	L	7.515	− 51	− 58	44
Angular gyrus	LTPJ	L	7.387	− 45	− 55	29
Supramarginal gyrus	LTPJ	L	7.349	− 63	− 46	23
Middle temporal gyrus	LTPJ	L	7.325	− 60	− 52	20
Angular gyrus	LTPJ	L	7.023	− 57	− 58	26
Right superior temporal sulcus (cluster size: 268)						
Middle temporal gyrus	RSTS	R	7.877	57	− 28	− 7
Superior temporal gyrus	RSTS	R	7.556	48	− 31	− 4
Middle temporal gyrus	RSTS	R	7.191	57	− 16	− 10
Middle temporal gyrus	RSTS	R	6.484	51	− 16	− 19
Middle temporal gyrus	RSTS	R	5.356	60	− 13	− 22
Precuneus (cluster size: 221)						
Posterior cingulate gyrus	PC	L	7.285	− 6	− 55	32
Posterior cingulate gyrus	PC	L	7.167	− 6	− 49	26
Right superior temporal sulcus (cluster size: 56)						
Temporal pole: middle temporal gyrus	RSTS	R	7.065	54	11	− 28
Temporal pole: middle temporal gyrus	RSTS	R	6.211	48	17	− 34
Temporal pole: middle temporal gyrus	RSTS	R	6.079	42	20	− 34
Right temporoparietal junction (cluster size: 68)						
Angular gyrus	RTPJ	R	6.928	60	− 55	29
Angular gyrus	RTPJ	R	5.727	51	− 58	35

*AAL*, automated anatomical labelling atlas; *MMPFC*, middle medial prefrontal cortex; *DMPFC*, dorsomedial prefrontal cortex; *VMPFC*, ventromedial prefrontal cortex; *LTPJ*, left temporoparietal junction; *RTPJ*, right temporoparietal junction; *RSTS*, right superior temporal sulcus; *PC*, precuneus; *L*, left; *R*, right

### Whole-Brain Analysis

We also performed a whole-brain analysis to explore activation outside of the ToM mask. We found activations in the temporal, parietal, lateral, and medial frontal cortex, anterior cingulate cortex, and thalamus to non-congruent vs. congruent images (see Supplementary material, Table S1). It is important to note that there was no significant gender difference in the activated brain regions (this analysis did not produce any significant voxels). In addition, contrasting congruent images versus non-congruent ones led to activation only in the lingual gyrus (see Supplementary material, Table S1).

### Sensitivity Analysis

One might argue that the difference in activation between non-congruent and congruent images could be due to the former capturing attention to a greater extent. To rule out this possibility, a total of 11 ROIs were combined into a mask to cover the dorsal frontoparietal network (also referred to as the dorsal attention system or visuospatial system), as described by Shirer et al. (2012). The resting-state functional ROIs were obtained from the website of the Greicius Laboratory at Stanford University. A small-volume correction (SVC) was implemented within the mask, resulting in increased activations in a small region (voxel size, 12; peak MNI coordinates x/y/z = − 54/14/23; *t*(1, 52) = 5.60 SVC, pFWE < .001) in response to non-congruent versus congruent images. This activation was located in the left frontal operculum. Given the relatively small size of the activation, it can be reasonably inferred that the two types of images did not recruit the dorsal frontoparietal network to a significantly different extent.

### Stimuli Validation

#### Participants and Procedure

This study involved university students who were compensated with extra course credits. A total of 48 participants initially began the assessment phase; however, four individuals decided to stop participating. The data from the remaining 44 participants (38 female, mean age = 21.89, *SD* = 3.27, age range = 19–40) were subsequently analysed. Each image was presented individually, accompanied by six questions on Qualtrics. The study was conducted with invited participants only, who received links to the study via their courses; there was no advertisement of the study on social media or other online platforms. The study was approved by the Institutional Review Board of Eötvös Loránd University, Faculty of Education and Psychology (approval number: 2024/462).

The aim of this study was to investigate whether there were any differences between congruent and non-congruent images. To address this, the rating dimensions were selected to align with the research question. Following the methodology of Langner et al. (2010), we evaluated the genuineness, intensity, and clarity of the expressed emotions, using a Likert scale ranging from 1 to 7. For a detailed description of the scales, see Table 4.

**Table 4:** Wording of items

Dimension	Instructions	Scale
1. Genuineness (based on Langner et al., 2010)	To what extent are the expressions displayed by the people in this image faked–genuine?	1 = *Faked*, 7 = *Genuine*
2. Surprising/strange	To what extent is this image surprising/strange?	1 = *Not at all strange/surprising* 7 = *Completely strange/surprising*
3. Intensity (based on Langner et al., 2010)	To what extent are the expression displayed by the people in this image weak–strong?	1 = *Very weak*, 7 = *Very strong*
4. Clarity (based on Langner et al., 2010)	To what extent are the expression displayed by the people in this image unclear–clear?	1 = *Very unclear*, 7 = *Very clear*
5. Mentalizing (based on the difference between the recognition of emotional expression vs. mentalizing—based on Barrett et al., 2019; Spunt & Adolphs, 2019)	In order to identify the emotional states of characters depicted in the image, is it sufficient to examine their facial expressions or postures, or must the entire context be taken into account?	1 = *It is enough to rely on the facial expressions or postures*, 7 = *Understanding the entire context is essential*
6. Understanding complexity	To what extent is easy-difficult to interpret the situation depicted in this image?	1 = *Very easy*, 7 = *Very difficult*

In addition to these ratings, three other questions were designed to assess the dimensions of surprising/strange, mentalizing, and understanding complexity. The rationale for including these three ratings was based on the hypothesis that the neural or behavioral responses to congruent and non-congruent images may arise from fundamental differences inherent to the images themselves. Specifically, it could be argued that non-congruent images capture attention more readily and require greater cognitive effort to interpret the emotions of the characters or to understand the broader context of the depicted scenario. To test this hypothesis, three additional questions were created, as outlined in Table 4.

### Statistical Analysis

A series of paired *t*-tests were conducted to test the differences between the two image types on the six dimensions. The Bonferroni correction for multiple test was applied (*p* = .05/6 = .008); thus, the significance level was set at *p* ≤ .008.

## Results

The ratings of the non-congruent and congruent images on three dimensions (strange/surprising, strength of emotional expressions, and understanding complexity) did not differ significantly. However, participants rated the emotional expression of protagonists in non-congruent images as less genuine and clear than in congruent images. They rated these images as requiring a more comprehensive understanding of the context (Table 5).

**Table 5:** Ratings in each dimension with the results of the paired *t*-tests

	Non-congruent	Congruent	*t*	*p*
Genuine	4.69 (1.14)	5.99 (0.82)	3.210	.001
Strange/surprising	3.28 (2.05)	3.34 (1.62)	0.078	.396
Strength of emotional expressions	4.97 (1.48)	5.06 (1.11)	0.168	.147
Clarity	4.66 (1.55)	5.08 (1.20)	0.742	.001
Mentalizing	4.92 (0.96)	4.21 (1.26)	0.153	.001
Understanding complexity	3.28 (1.79)	3.10 (1.29)	0.273	.032^*^

Note. ^*^Not significant after Bonferroni correction of *p* values (significance level was set at *p* ≤ .008)

## Discussion

Our primary objective was to investigate whether specific situations necessitate a greater reliance on ToM than others. Our findings are consistent with the theoretical model proposed by Bird and Viding (2014), showing that the ToM system is more engaged when there is a mismatch between affective cues and situational information. In other words, this increased engagement occurs when understanding the emotional state of a target individual cannot be achieved through affective cues alone (i.e., affective ToM) but requires consideration of the target’s motives, thoughts (i.e., cognitive ToM), or the emotional and cognitive states of others around them. Using a pre-defined ToM mask, we observed increased activations in all the core regions typically associated with ToM, including the ventromedial and dorsomedial prefrontal cortex, middle medial prefrontal cortex, precuneus, bilateral temporoparietal junctions, and the right superior temporal sulcus. These findings align with the theory proposed by Saxe and Houlihan (2017), which posits that emotional responses to stimuli in context are based on forward inferences. Our study demonstrated that this process of inference recruits the ToM system more prominently when emotion attribution would yield significantly different outcomes without considering the contextual information. It is important to note that we classified images as non-congruent when the valence of the facial expression of (at least) one of the protagonists was somewhat opposite to the overall context. This approach differs from studies utilizing artificial scenes, where the context was somewhat independent of the target’s facial expression (Ngo & Isaacowitz, 2015). By contrast, our study focuses on naturalistic emotional scenes in which context plays a crucial role in shaping the emotional interpretation of facial expressions.

In response to the question of whether specific situations require a greater reliance on ToM than others (Bio et al., 2018; Conway & Bird, 2018), our findings suggest that the understanding of certain complex social situations is more reliant on the ToM system. Specifically, we observed that the processing of non-congruent images results in a more pronounced activation of the ToM system. However, it is essential to determine whether the two categories of images, designated as either congruent or non-congruent, exhibited any distinguishing characteristics that could explain the observed outcomes. Our study revealed no significant difference in the number of individuals (two or more) depicted in the image types. Nevertheless, it is possible that the amount of social information to process may differ between congruent and non-congruent images. For instance, one study demonstrated that the amount of social information, referred to as social load, when making judgments about others, influenced the extent of recruitment of the dorsomedial prefrontal cortex, precuneus/posterior cingulate cortex, and TPJ—regions that are implicated in mentalizing (Meyer et al., 2012). Similarly, it would be valuable to investigate whether there is an additional demand on social working memory resources when processing non-congruent images. This consideration aligns with previous research on the processes underlying judgments about others. In that research, the selection of social information from competing alternatives, as opposed to the controlled retrieval of social information, resulted in greater involvement of the dorsomedial prefrontal cortex and the TPJ (Satpute et al., 2014).

In examining the behavior of others, one may choose to focus on various aspects. For example, one might observe the action itself (e.g., smiling) but also consider the underlying motivation behind that action (e.g., pride) (Spunt & Adolphs, 2014; Spunt et al., 2011). In the latter case, we attribute to the observed individual an intention, emotion, belief, or goal—in other words, an unobserved mental state. It seems reasonable to posit that, in this context, where the question is “why” the behavior is happening rather than “how” it is happening, the ToM system is activated. Indeed, research has shown that focusing on why a social behavior is occurring modulates brain responses and recruits the ToM system, in contrast to when the question concerns how something is happening (Spunt & Adolphs, 2014). Our results raise the question of how the activation of the ToM system for “why” questions is modulated by the nature of the context.

To gain further insight into the neural response distinction between non-congruent and congruent images, a stimuli validation study assessed the images across a range of dimensions, with participants who were not involved in the fMRI study. For non-congruent images, participants were more likely to report that they relied on mentalizing to a greater extent. In other words, they indicated that understanding the entire context was more essential for these images, although both means were above the nominal midpoint of the scale (4.92 vs. 4.21 on the 7-point Likert scale). Participants rated the situations depicted in both image types as equally straightforward to interpret (understanding complexity). This finding suggests that even non-congruent situations were perceived as natural as congruent ones. Similarly, no significant difference was observed between the two image types with respect to strangeness or surprise. Furthermore, the intensity of the expression displayed by the character in both image types was rated as identical. However, the protagonists of non-congruent images were rated as expressing emotions less clearly and less genuinely than those in congruent images. Despite this, the expressions in both image types were considered fairly genuine and clear, with means exceeding the nominal midpoint of the scale. Notably, non-congruent negative images predominantly depicted situations in which a smiling facial expression was perceived as negative within the context, such as instances of bullying or fighting. Consequently, it is possible that participants did not consider the smile itself to be a genuine and clear expression of the underlying emotion (e.g., hatred).

Given that the core regions of the ToM network are, in fact, part of the default mode network (DMN), discussing the role of the DMN in emotion perception and experience (Satpute & Lindquist, 2019) provides a useful framework for interpreting our findings. According to the theory of constructed emotion (Barrett, 2017), the ability to identify discrete emotions, either in oneself or others, depends on many prior and variable instances of that specific emotion, such as sadness. In a given situation, we draw upon these past experiences and semantic knowledge to generate predictions about the meaning of internal or external experiences and behaviors. In other words, the perception of sadness is contingent upon the convergence of perceptual, behavioral, and contextual information that aligns with our individualized conceptualization of sadness. This process, known as conceptualization, is facilitated by the DMN, as proposed by Satpute and Lindquist (2019). If the DMN is indeed responsible for this process, it follows that emotional stimuli requiring greater conceptualization should activate the DMN to a greater extent (Satpute & Lindquist, 2019). In light of this framework, it seems reasonable to hypothesize that the images labelled “non-congruent” in this study necessitated a greater degree of conceptualization, particularly at the abstract level of situated conceptualization of an emotion category.

Although there was no difference in low visual complexity indices between non-congruent and congruent images, and the participants of the stimuli validation study did not rate them as more strange or surprising, it is possible that non-congruent images may still capture attention to a greater extent at the neural level. Accordingly, we investigated whether processing non-congruent images, compared to congruent images, engaged the dorsal frontoparietal network, which is implicated in visual attentional processes (Corbetta et al., 2002; Long & Kuhl, 2018), to a greater extent. The magnitude of the observed activation was relatively modest, indicating that the two types of images did not elicit a significantly different degree of recruitment of the dorsal frontoparietal network. The absence of differences in the involvement of this network, in conjunction with the findings of the stimuli validation study, suggests that non-congruent images did not capture attention to a greater extent than congruent images. This lack of difference may be, at least in part, attributable to the fact that both types of images are socially relevant visual stimuli depicting everyday or easily understandable life situations. Additionally, in the post-task ratings, participants in the fMRI study rated non-congruent and congruent images as equally arousing. Regarding valence ratings, congruent positive images were rated slightly more positively than non-congruent positive images; however, both types were rated as highly pleasant. Finally, reaction times to the response screen indicated that participants responded more slowly during trials involving negative and non-congruent stimuli, compared to the other three types of images. This finding is consistent with our previous results using a subsample from this study (Biro et al., 2022).

Testing the activations in the dorsal frontoparietal system is also relevant for the recruitment of TPJ in social cognition studies. The mask used for our analysis included the bilateral temporoparietal junction. However, there is an ongoing debate regarding the involvement of the TPJ in ToM, specifically whether the (r)TPJ selectively subserves mentalizing processes, such as belief attribution. The debate centres around whether TPJ activation during mentalizing tasks reflects its role in mental state attribution (Saxe & Wexler, 2005), given that this region is also recruited in tasks requiring the reorientation of attention (Mitchell, 2008). Meta-analytic evidence, however, suggests that the TPJ is involved in both processes (Krall et al., 2015), raising further questions. If the TPJ is implicated in both attention reorientation and mentalizing, does this indicate that distinct subregions are responsible for these functions, or is there a cognitive mechanism that allows the TPJ to support both roles? Concerning the subregional hypothesis, a meta-analysis of task-based fMRI data and resting-state functional connectivity data (Krall et al., 2015) suggests that the anterior and posterior parts of the rTPJ are associated with mentalizing and attention reorientation in different ways. Specifically, the anterior section may be involved in both processes, while the posterior section seems to be exclusively associated with ToM, particularly in false belief tasks. Moreover, the specific task used to assess mentalizing could influence TPJ recruitment. For instance, a study by Atique et al. (2011) found that when participants were asked to infer either emotion or intention in others, emotion mentalization and intention mentalization activated distinct subregions of the right and left TPJ. Notably, the anterior TPJ region, which showed greater activation for emotion mentalization compared to intention mentalization, demonstrated stronger functional connectivity with other components of the mentalizing network, particularly the ventromedial prefrontal cortex (VMPFC). This suggests that inferring beliefs, emotions, or intentions may engage different subregions of the TPJ.

Regarding the proposition that a cognitive mechanism might explain the TPJ’s involvement in both attention and mentalizing, it has been proposed that the TPJ contributes to contextual updating (Geng & Vossel, 2013) or, more generally, to predictive processing (Masina et al., 2022). In the case of non-congruent images, emotions predicted from a given situation may differ from those inferred from the facial expressions of individuals within that context. Thus, updating the social environment model and forming predictions about another person’s behavior are more critical in this case than in images where perceptual and conceptual information are not in conflict. Alternatively, our findings may be interpreted within the framework suggesting that TPJ involvement in mentalizing reflects the integration of multiple processes that contribute to understanding others’ mental states (Schaafsma et al., 2015).

It is important to explicitly state that the task was not originally designed to capture neural responses to congruent and non-congruent emotional stimuli. Consequently, the same character with the same facial expression was not presented twice with different (congruent and non-congruent) backgrounds. Additionally, it is plausible that the internal structure of the Emotional Situation Task (EST) made participants more aware of the varying valence between the Emotion1 and Emotion2 image pairs in the non-congruent images. This awareness may have subsequently influenced the activation of the ToM network and other brain regions. Therefore, we cannot be certain that the same results would have been obtained if only Emotion2 images were used. Future studies could examine whether the same results could be replicated using only the full (Emotion2) images. Furthermore, a comparison with the findings from the previous publication, which focused on the contrast of Emotion2 > Emotion1 images in the EST, supports this conclusion. Specifically, a shift in valence was found to activate certain regions of the ToM network, including the TPJ and precuneus. Notably, the Emotion2 images were categorized as non-congruent based on the opposing valence between the Emotion1 and Emotion2 images. This limitation should be considered when interpreting the findings related to the ToM system, as well as the result of a whole-brain analysis discussed below.

A whole-brain analysis found increased activation outside of our pre-defined ToM mask in various regions, including the temporal, parietal, lateral, and medial frontal cortex, anterior cingulate cortex, and thalamus. Activation of salience network areas (Tholen et al., 2020) along with the thalamus and supplementary motor area is consistent with previous empathy studies (Bzdok et al., 2012). They are also implicated in affective processing (Kohn et al., 2014; Sambuco et al., 2020), indicating that evaluation of others’ internal state in the so-called non-congruent images vs. congruent ones is supported by affective processing.

Increased activations in the anterior and posterior frontoparietal control network (FPCN) subsystems were also observed. Dixon et al. (2018) proposed that these subsystems might have distinct roles in executive functioning. Specifically, the anterior FPCN, connected with the default network, may regulate thoughts and emotions, supporting social reasoning. Conversely, the posterior subsystem, linked with the dorsal attention system, likely regulates perceptual attention. Furthermore, the role of dlPFC in regulating the valence of emotional experience has recently been demonstrated (Nejati et al., 2021). Our analysis revealed increased activations in both subsystems when processing images with non-congruent information, implying that understanding such situations demands increased executive control processes to facilitate accurate mentalization or matched empathic responding. This aligns with the results of effective connectivity studies using social cognition tasks (Schurz et al., 2020), where increased positive coupling between control networks and default mode network (e.g., mPFC areas in our ToM mask) reflects more effortful and controlled processing.

Outside of our pre-defined mask, we found increased activation in IFG and orbitofrontal areas, which are known to be involved in perceiving emotional expressions (Belyk et al., 2017). Furthermore, compared to control subjects, people with lesion in the orbitofrontal cortex (OFG) performed worse in affective ToM task compared to cognitive ToM task (Shamay-Tsoory et al., 2010), and OFC has been shown to predict more accurate valence evaluation for high ambiguity stimuli in a study using scene images of naturalistic social interactions (Deuse et al., 2016). Therefore, increased OFG activation in our study may support the view (Belyk et al., 2017) that inferior OFG plays a particular role in evaluating the emotional value of stimuli.

We did not find any sex differences in the activations to non-congruent vs. congruent images. Previous studies investigating gender differences in empathy and ToM tasks have applied different methodologies (Belyk et al., 2017; Paracampo et al., 2018; Schulte-Ruther et al., 2008). Our task did not explicitly ask participants to categorize targets’ emotional states, which might account for the absence of sex differences in our study.

## Limitation

One limitation is that the EST does not involve a fixation interval after Emotion2 images, which raises the possibility that the activation to these images is contaminated with the activation to smileys/emojis. However, we believe that a presentation time of a least 8 s is sufficient to prevent contamination by subsequent responses. In a subsequent study with EST, we advise including a fixation interval between Emotion2 and the smiley screen. Additionally, we did not measure trait empathy, which prevented us from testing how inter-individual differences in different facets of empathy, such as personal distress, empathic concern, or perspective-taking (Davis, 1983; Kogler et al., 2020), might relate to neural response to non-congruent vs. congruent images. For example, a study found that empathic accuracy during emotional videos was related to self-reported perspective-taking and activity of brain regions (STS, TPJ, and temporal pole) implicated in cognitive empathy or ToM (Mackes et al., 2018).

The participants were instructed to simply observe the images and attempt to become emotionally engaged with the depicted scenarios. However, modifying the instructions for the Emotional Situation Task (EST) could be a valuable improvement. For instance, asking participants to identify the overt behaviors (e.g., smiling) or the potential causes of these behaviors (e.g., “having fun with friends” or “enjoying teasing others”) might engage different neural systems. In the former case, the mirror system would likely be activated, whereas in the latter, the ToM system would be more prominently engaged (Spunt & Lieberman, 2012). Additionally, a non-social version of this task could help clarify the ongoing debate about the role of the TPJ in mentalizing. For example, an aesthetically pleasing chair could be placed in a visually unappealing room (a non-congruent image) or in a room that is pleasing to the eye (a congruent image).

A thorough analysis of the EST images revealed that the two image types were not perfectly counterbalanced in terms of discrete emotions, including anger, fear, sadness, and happiness. Consequently, to eliminate the possibility that discrete emotions influenced the activation of the ToM network, further refinement of the task is necessary. However, there is no evidence to suggest that specific emotions recruit the ToM network to a greater extent than others. Therefore, it can be assumed that the observed differences are not attributable to discrete emotions. Furthermore, there is no consistent evidence linking specific emotions to distinct brain regions (Lindquist et al., 2012). This supports the argument that the observed differences in our study are not due to discrete emotion categories.

## Conclusion

In conclusion, our results suggest that understanding the behaviors of others and adjusting our own behavior accordingly require the representation of both our own and others’ mental states (Kanske, 2018), which are closely tied to the context in which the behavior is perceived (Saxe & Houlihan, 2017). We found increased recruitment of the ToM network in complex situations, where emotion attribution without considering the scene context would lead to significantly different interpretations, compared to situations where the emotional outputs would be more aligned. However, whether the heightened activation of the ToM system in response to non-congruent images reflects improved mentalizing ability and more accurate empathic responses remains unclear. Further research is needed to explore how and when the ToM system contributes to precise emotional processing and social perception in intricate social scenarios. Additionally, the increased activation of emotion processing and control areas in response to non-congruent images, as compared to congruent ones, suggests that processing complex social situations involves multiple cognitive processes. Therefore, identifying the sources of alterations in emotion processing and social cognition is crucial for understanding individual differences in both neurotypical and clinical populations.

## Supplementary Information

Below is the link to the electronic supplementary material.

Supplementary file1 (DOCX 33 kb)

## Additional Information

### Data Availability

The datasets generated and/or analysed during the current study are not publicly available as we cannot share the raw fMRI data publicly, because at the time our study started, there was no information on open access data availability in the consent, but raw data are available from the corresponding author on reasonable request. However, main fMRI contrast maps are fully available at https://osf.io/zqwub/.
