# Supplementary material for: Unveiling the Role of Theory of Mind: Neural Response to Emotional Stimuli in Context
Source: Affect Sci. 2025 Feb 26;6(2):340–55. doi: 10.1007/s42761-025-00293-1 (PMC12209149; doi:10.1007/s42761-025-00293-1)
Supplement: Supplementary file 1 — Supplementary file1 (DOCX 33 kb) [file 42761_2025_293_MOESM1_ESM.docx]

**Unveiling the Role of Theory of Mind: Neural Response to Emotional Stimuli in Context**

Brigitte Biró^1,2,3,4^, Renáta Cserjési^3^, Natália Kocsel^3^, Attila Galambos^1,3^, Kinga Gecse^2,5,6^, Lilla Nóra Kovács^3^, Dániel Baksa^2,5,7^, Dóra Dobos^2,5,6^, Gabriella Juhász^2,5^ Gyöngyi Kökönyei^2,3,5*^

^1^Doctoral School of Psychology, ELTE Eötvös Loránd University, Budapest, Hungary

^2^NAP3.0-SE Neuropsychopharmacology Research Group, Hungarian Brain Research Program, Semmelweis University, Budapest, Hungary

^3^ Institute of Psychology, ELTE Eötvös Loránd University, Budapest, Hungary

^4^ Department of Clinical Psychology, Semmelweis University, Budapest, Hungary.

^5^ Department of Pharmacodynamics, Faculty of Pharmaceutical Sciences, Semmelweis University, Budapest, Hungary

^6^ PhD Scholarship, Doctoral School of Mental Health Sciences, Semmelweis University, Budapest, Hungary

^7^ Department of Personality and Clinical Psychology, Institute of Psychology, Faculty of Humanities and Social Sciences, Pazmany Peter Catholic University, Budapest, Hungary

Table S1. Increased activations to non-congruent vs. congruent images (FWE corrected level of p < .05 at a whole brain level).

| **Cluster size (voxel)** | **Region** | **Br** | **Hemi-sphere** | **Peak T-values** | **MNI coordinates** | | |
| --- | --- | --- | --- | --- | --- | --- | --- |
|  |  |  |  |  | x | y | z |
| Non-congruent images > Congruent images | | | | | | | |
| 972 | Middle temporal gyrus |  | L | 8.80 | -60 | -37 | -1 |
|  | Angular gyrus |  | L | 8.11 | -42 | -64 | 44 |
|  | Angular gyrus |  | L | 7.62 | -51 | -55 | 32 |
|  | Angular gyrus |  | L | 7.39 | -45 | -55 | 29 |
|  | Middle temporal gyrus |  | L | 7.33 | -60 | -52 | 20 |
|  | Middle temporal gyrus |  | L | 7.32 | -63 | -13 | -16 |
|  | Middle temporal gyrus |  | L | 7.12 | -63 | -25 | -10 |
|  | Angular gyrus | 39 | L | 7.02 | -57 | -58 | 26 |
|  | Middle temporal gyrus |  | L | 6.71 | -51 | -22 | -7 |
|  | Inferior parietal gyrus |  | L | 6.67 | -57 | -49 | 41 |
|  | Inferior parietal gyrus |  | L | 6.64 | -54 | -49 | 47 |
|  | Middle temporal gyrus |  | L | 6.54 | -54 | -19 | -19 |
|  | Middle temporal gyrus | 21 | L | 6.46 | -54 | 8 | -28 |
|  | Middle temporal gyrus | 21 | L | 6.21 | -54 | -1 | -28 |
|  | Middle temporal gyrus |  | L | 5.80 | -42 | 2 | -31 |
| 1234 | Superior Frontal Gyrus, medial |  | L | 8.12 | -6 | 59 | 11 |
|  | Anterior cingulate cortex, pregenual | 24 | L | 7.48 | -3 | 38 | 8 |
|  | Anterior cingulate cortex, pregenual |  | L | 7.07 | -3 | 50 | 8 |
|  | Superior frontal gyrus, medial |  | R | 6.66 | 9 | 56 | 26 |
|  | Superior frontal gyrus, medial | 8 | L | 6.49 | -6 | 41 | 50 |
|  | Anterior cingulate cortex, subgenual | 32 | R | 6.48 | 3 | 35 | -4 |
|  | Middle frontal gyrus |  |  | 6.39 | -24 | 47 | 32 |
|  | Supplementary motor area |  | R | 6.36 | 6 | 14 | 65 |
|  | Superior frontal gyrus, medial |  | L | 6.31 | -9 | 56 | 26 |
|  | Superior frontal gyrus, medial orbital | 10 | L | 6.10 | -3 | 53 | -7 |
|  | Superior frontal gyrus, medial |  | L | 6.01 | -9 | 26 | 59 |
|  | Supplementary motor area | 6 | L | 5.99 | -3 | 14 | 62 |
|  | Supplementary motor area |  | L | 5.98 | 0 | 5 | 68 |
|  | Anterior cingulate cortex, supracallosal | 32 | L | 5.95 | -3 | 35 | 23 |
| 282 | Middle temporal gyrus |  | R | 7.88 | 57 | -28 | -7 |
|  | Superior temporal gyrus |  | R | 7.56 | 48 | -31 | -4 |
|  | Middle temporal gyrus | 21 | R | 7.19 | 57 | -16 | -10 |
|  | Inferior Temporal Gyrus |  | R | 6.48 | 51 | -16 | -19 |
|  | Middle temporal gyrus |  | R | 5.36 | 60 | -13 | -22 |
|  | Inferior temporal gyrus | 21 | R | 5.18 | 57 | -25 | -19 |
| 247 | Posterior cingulate gyrus |  | L | 7.29 | -6 | -55 | 32 |
|  | Posterior cingulate gyrus |  | L | 7.17 | -6 | -49 | 26 |
| 137 | Inferior frontal gyrus, pars orbitalis |  | L | 7.21 | -48 | 23 | -7 |
|  | Inferior frontal gyrus, triangular part | 45 | L | 6.03 | -54 | 26 | 14 |
|  | Temporal pole: superior temporal gyrus |  | L | 5.89 | -36 | 17 | -22 |
| 60 | Temporal Pole: middle temporal gyrus |  | R | 5.89 | 54 | 11 | -28 |
|  | Temporal Pole: middle temporal gyrus |  | R | 5.35 | 48 | 17 | -34 |
|  | Temporal Pole: middle temporal gyrus | 38 | R | 5.26 | 42 | 20 | -34 |
| 68 | Angular gyrus | 40 | R | 6.93 | 60 | -55 | 29 |
|  | Angular gyrus |  | R | 5.73 | 51 | -58 | 35 |
| 89 | Middle frontal gyrus |  | L | 6.16 | -36 | 44 | 5 |
|  | Middle frontal gyrus |  | L | 6.13 | -42 | 47 | 8 |
|  | Inferior Frontal gyrus, triangular part | 46 | L | 5.59 | -51 | 41 | 5 |
| 11 | Thalamus |  | R | 5.80 | 6 | -25 | 2 |
| 25 | Middle cingulate gyrus |  | L | 4.98 | 0 | -16 | 35 |
| 18 | Inferior frontal gyrus, opercular part |  | L | 5.60 | -54 | 14 | 23 |
| 21 | Middle frontal gyrus |  | L | 5.47 | -42 | 14 | 38 |
| 3 | Amygdala |  | R | 5.40 | 24 | -7 | -13 |
| 5 | Caudate |  | L | 5.30 | -12 | 14 | 2 |
| 6 | Hippocampus |  | L | 5.24 | -24 | -19 | -13 |
|  | Hippocampus |  | L | 5.18 | -21 | -10 | -16 |
| Congruent images > Non-congruent images | | | | | | | |
| 52 | Lingual gyrus |  | L | 5.77 | -18 | -79 | -7 |
|  | Lingual gyrus |  | L | 5.33 | -9 | -85 | -10 |

Note: Br: Broadmann Area; L: left; R: right
